# 29. THE COMPLEX INTERACTIONS BETWEEN MIND AND BODY: IT TAKES A BRAIN TO CONTROL IMMUNITY

**DOI:** 10.1093/schbul/sby014.120

**Published:** 2018-04-01

**Authors:** Asya Rolls

**Affiliations:** Israel Institute of Technology

## Abstract

**Overall Abstract:** Thoughts and emotions can impact physiology. This connection is evident by the emergence of disease following stress or recovery in response to placebo treatment. Nevertheless, this fundamental aspect of physiology remains largely unexplored. We have recently shown that activation of the brain’s reward system, which is active in positive emotional states and positive expectations, boosts immunity. In this talk, I will discuss how brain activity can regulate anti-bacterial and anti-tumor immunity and the potential implications for health and cancer therapy. Given the crucial role of the reward system in emotional processes, our findings offer a new mechanistic insight to the association between the patient’s psychological state, physical health and cancer progression.

